# An inexact fractional programming model for irrigation water resources optimal allocation under multiple uncertainties

**DOI:** 10.1371/journal.pone.0217783

**Published:** 2019-06-13

**Authors:** Chongfeng Ren, Jiantao Yang, Hongbo Zhang

**Affiliations:** 1 Key Laboratory of Subsurface Hydrology and Ecological Effect in Arid Region, Ministry of Education, Xi’an, Shaanxi Province, China; 2 School of Environmental Science and Engineering, Chang’an University, Xi’an, Shaanxi Province, China; Shandong University of Science and Technology, CHINA

## Abstract

In reality, severe water shortage crisis has made bad impact on the sustainable development of a region. In addition, uncertainties are inevitable in the irrigation system. Therefore, a fully fuzzy fractional programming model for optimization allocation of irrigation water resources, which aimed at not only irrigation water optimization but also improving water use efficiency. And then the developed model applied to a case study in Minqin County, Gansu Province, China, which selected maximum economic benefit of per unit water resources as planning objective. Moreover, surface and underground water are main water sources for irrigation. Thus, conjunctive use of surface and underground water was taken under consideration in this study. By solving the developed model, a series of optimal crop area and planting schemes, which were under different *α*-cut levels, were offered to the decision makers. The obtained results could be helpful for decision makers to make decision on the optimal use of irrigation water resources under multiple uncertainties.

## Introduction

Today, water resources scarcity, which has great negative influence on the development of society and economy, becomes more and more serious [1–3]. Moreover, the demand for water resources is growing rapidly because of the rapid growth of economy and society [4–6]. Thus, there is great conflict between the growing demand for water resources and limited water resources. However, irrigation consumes approximately 70% of the world’s freshwater resources [7]. Especially in the arid and semi-arid regions of Northwest China, characterized with high evaporation and low rainfall, approximately 90% of freshwater resources has been used to irrigation [7–10]. Therefore, optimization allocation of irrigation water resources has great positive influence on the sustainable development of a region.

Over the past decades, there were a series of studies about optimization allocation of irrigation water resources [11–20]. The above studies were mainly focused on maximizing yield, maximizing economic benefit, balancing between multi-objectives, or minimizing system cost. However, the more output (yield, economic benefit) means that more water is need for irrigation; while there is no more water for irrigation under the severe water resources shortage situation. Therefore, the core of irrigation water optimization allocation is water use efficiency, i.e., output of unit water resources. Moreover, in reality, decision-makers are paying more and more attention to the output of unit water resources, which can be used as an effective indicator for measuring water resources consumption [21,22]. Fractional programming can deal with the above problems effectively [23,24]. It can make sure relatively more output by consuming unit of water resources because the objective function of fractional programming is the ratio of output to input [25]. Moreover, the decision makers can also improve unreasonable plans based on the optimal schemes under the optimal ratio.

In reality, irrigation system is a relatively complex system, which involves many aspects of economy and society. Thus, uncertainties are inevitable in the operation of optimizing irrigation water resources, such as random, fuzzy and interval [26–28]. In the past, many studies have been carried out about optimization allocation of irrigation water resources under certainty. For example, an inexact stochastic dynamic programming model, which aimed to maximize the total economic benefit, was established for water resources management by Gu [29]. Borgomeo et al. [30] presented a risk approach for incorporating nonstationary probabilistic climate projections into long-term water resources planning. In order to achieve sustainable development of agriculture, a two-level optimization model was developed, which combined use of SWAP-EPIC model [31]. Liu et al. [32] presented a Monte Carlo simulation based dual-interval stochastic programming for optimizing crop planning, which made maximum economic benefit as objective. When facing multi-water source for irrigation, an interval two-stage stochastic robust programming was developed for optimizing crop planning [33]. Although uncertainty was taken under consideration in the above studies, only one parameter with uncertainty was considered; or one or two kinds of uncertainties was considered. However, in reality, many parameters involve uncertainties, such as crop area, available surface water, available groundwater [34]. Furthermore, some parameters may change the characteristics of their uncertainty because of the effects of human activities. For example, runoff has random uncertainty under natural conditions; while it may have fuzzy uncertainty instead of random uncertainty when a lot of water resources is taken away. Therefore, this paper takes the above problems under consideration.

Therefore, this paper developed a fully fuzzy fractional programming model (FFFPM) for optimizing irrigation water resources under multiple uncertainties. This paper took food security, groundwater exploitation and available surface water under consideration. Then the presented model was applied to Minqin County, Gansu Province, China, which made maximum economic benefit of per unit water resources as objective function. Moreover, irrigation water resources were optimized by conjunctive use of groundwater and surface water simultaneously. A range of water resources optimal allocation schemes were provided based on the result of the established model. The developed model can be used to help decision makers identify a desired plan for optimizing irrigation water resources by conjunctive use of groundwater and surface water under multiple uncertainties.

## Model formulation

### Linear fractional programming

The general form of linear fractional programming is as following:

$$Max\;f\text{(}x\text{)}=\frac{Cx+\alpha }{Dx+\beta }$$ (1)

Ax≤B,x≥0 (2)

Where, *A* represents matrix with *m* row vectors and *n* column vectors; *B* and *x* represent the column vectors with *n* and *m* elements, respectively; *C* and *D* represent the row vector with n elements, respectively; and *α* and *β* means constant, respectively. Based on the assumption, *Dx* + *β* is constant for all *x* in the whole feasible region, by Charnels and Cooper, the linear fractional programming can be solved by solving the linear programming model [35].

According to Chadha, if the following can be satisfied: (1) all the *x* can meet *Dx* + *β* > 0; (2) the objective function is continuously differentiable; (3) the feasible is not empty and bounded, the linear fractional programming Eqs (1) and (2) can be transformed into Eqs (5)–(9) based on the Charnes-Cooper method, which introduces Eqs (3) and (4) into Eqs (1) and (2) [36,37].

$$Z=\frac{1}{Dx+\beta }$$ (3)

Y=Zx (4)

Therefore, the linear fractional programming model transformed into linear programming model, which presented as following:

Maxf(x)=CY+αZ (5)

AY≤BZ (6)

DY+βZ=1 (7)

Y≥0 (8)

Z>0 (9)

Therefore, the above linear fractional programming model Eqs (1) and (2) can be transformed into the linear programming model Eqs (5)–(9) by introducing intermediate variable Eqs (3) and (4). The linear fractional programming model has great advantage of dealing with the optimal ratio problem.

## Fully fuzzy linear fractional programming

In reality, a large amount of parameter in irrigation system has characteristics of fuzzy uncertainty. And the parameters with fuzzy uncertainty exist not only in constraints but also in objective functions. The form of FFFPM is as following:

$$Max\tilde{Z}=\frac{\sum_{j=1}^{n}{\tilde{c}}_{j}\cdot {\tilde{x}}_{j}+\tilde{p}}{\sum_{j=1}^{n}{\tilde{d}}_{j}\cdot {\tilde{x}}_{j}+\tilde{q}}$$ (10)

Subjective to:

$$\sum_{j=1}^{n}\sum_{i=1}^{m}{\tilde{a}}_{ij}\cdot {\tilde{x}}_{j}\leq {\tilde{b}}_{i},i=1,2,\ldots ,m$$ (11)

$${\tilde{x}}_{j}\geq 0,j=1,2,\ldots ,n$$ (12)

Where, ${\tilde{a}}_{ij}$ represents matrix with *j* row vectors and *i* column vectors with fuzzy uncertainty. ${\tilde{c}}_{j}$ and ${\tilde{d}}_{j}$ are row vector with fuzzy uncertainty for each *j* = 1, …,*n*, respectively. ${\tilde{x}}_{j}$ represents column vector with fuzzy uncertainty for each *j* = 1, …, *n*. $\tilde{p}$ and $\tilde{q}$ means constant with fuzzy uncertainty, respectively. $\tilde{b}$ is row vector with fuzzy uncertainty for each *i* = 1, 2, …, *m*.

**Fig 1:**
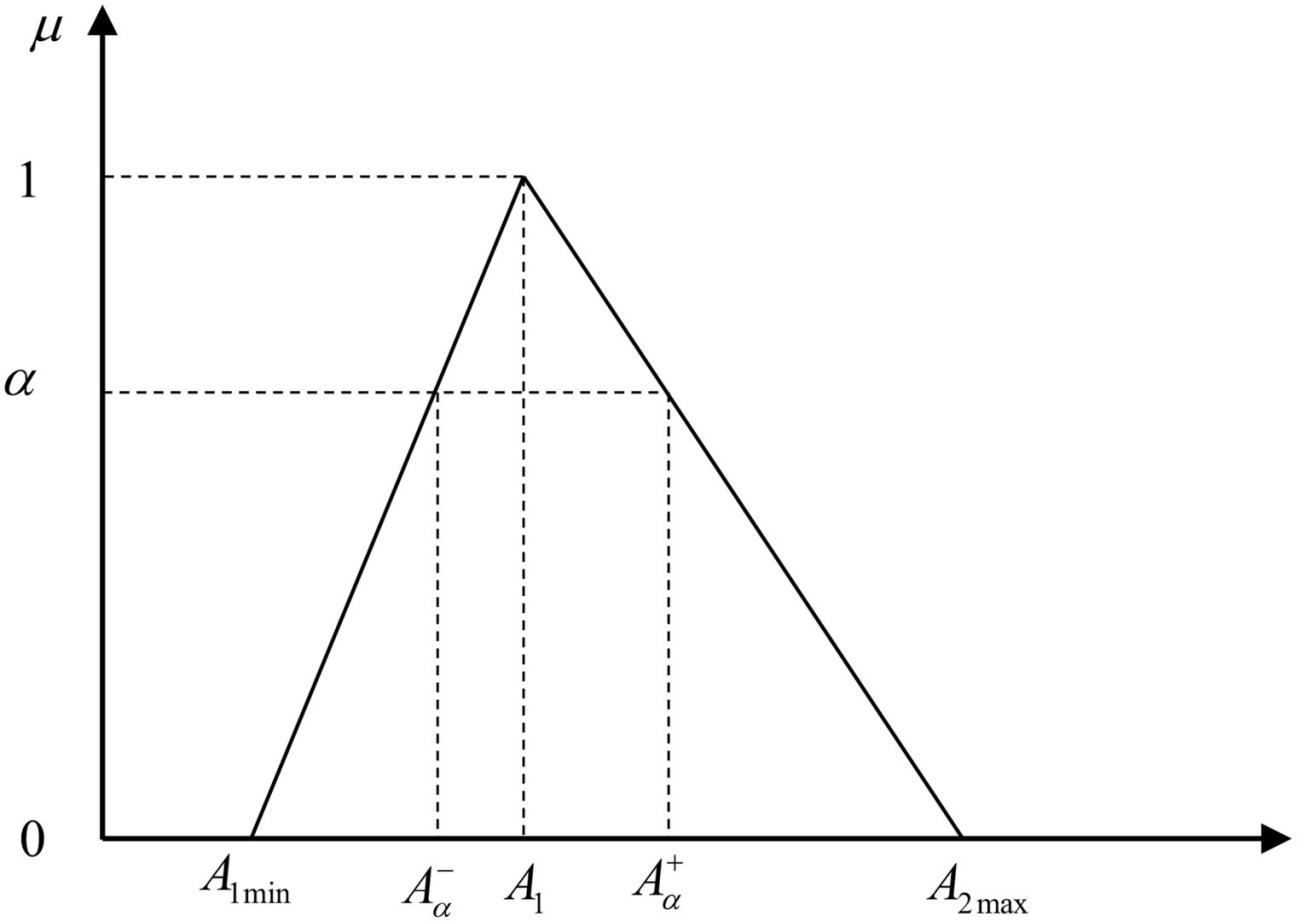
Triangle membership function.

In order to deal with the fuzzy fractional programming, fuzzy set theory was introduced which assume the parameters with fuzzy uncertainty are triangular fuzzy function as shown in Fig 1 [38].

The solving steps of FFFPM are as following:

Based on the assumption, ${\tilde{c}}_{j}$, $\tilde{p}$, $\tilde{q}$, ${\tilde{d}}_{j}$, $\tilde{b}$, ${\tilde{a}}_{ij}$ and ${\tilde{x}}_{j}$ are triangular fuzzy numbers which can represent as $\left(c_{j}^{1},c_{j}^{2},c_{j}^{3}\right)$, $\left(p_{j}^{1},p_{j}^{2},p_{j}^{3}\right)$, (*q*_1_, *q*_2_, *q*_3_), $\left(d_{j}^{1},d_{j}^{2},d_{j}^{3}\right)$, $\left(b_{i}^{1},b_{i}^{2},b_{i}^{3}\right)$, $\left(a_{ij}^{1},a_{ij}^{2},a_{ij}^{3}\right)$ and $\left(x_{j}^{1},x_{j}^{2},x_{j}^{3}\right)$, respectively.

Then, the FFFPM Eqs (10)–(12) can be written as follows:

$$Max\tilde{Z}=\frac{\sum_{j=1}^{n}\left(c_{j}^{1},c_{j}^{2},c_{j}^{3})\cdot (x_{j}^{1},x_{j}^{2},x_{j}^{3}\right)+(p^{1},p^{2},p^{3})}{\sum_{j=1}^{n}\left(d_{j}^{1},d_{j}^{2},d_{j}^{3}\right)\cdot (x_{j}^{1},x_{j}^{2},x_{j}^{3})+(q^{1},q^{2},q^{3})}$$ (13)

Subject to

$$\sum_{j=1}^{n}\sum_{i=1}^{m}\left(a_{ij}^{1},a_{ij}^{2},a_{ij}^{3})\cdot (x_{j}^{1},x_{j}^{2},x_{j}^{3}\right)\leq (b_{i}^{1},b_{i}^{2},b_{i}^{3}),\;i=1,\ldots ,m$$ (14)

$$(x_{j}^{1},x_{j}^{2},x_{j}^{3})\geq 0,\;j=1,2,\ldots ,n$$ (15)

Then, setting different *α*-cut levels for the parameters among objective function and constraints based on the fuzzy set theory. The above Eqs (13)–(15) can be written as follows:

$$Max\tilde{Z}=\frac{\begin{array}{l} \sum_{j=1}^{m}\left[c_{j}^{1}+(c_{j}^{2}-c_{j}^{1})\cdot \alpha ,c_{j}^{3}-(c_{j}^{3}-c_{j}^{2})\cdot \alpha \right]\cdot \left[x_{j}^{1}+(x_{j}^{2}-c_{j}^{1})\cdot \alpha ,x_{j}^{3}-(x_{j}^{3}-x_{j}^{2})\cdot \alpha \right]\\ +\left[p_{j}^{1}+(p_{j}^{2}-p_{j}^{1})\cdot \alpha ,p_{j}^{3}-(p_{j}^{3}-p_{j}^{2})\cdot \alpha \right] \end{array}}{\begin{array}{l} \sum_{j=1}^{m}\left[d_{j}^{1}+(d_{j}^{2}-d_{j}^{1})\cdot \alpha ,d_{j}^{3}-(d_{j}^{3}-d_{j}^{2})\cdot \alpha \right]\cdot \left[x_{j}^{1}+(x_{j}^{2}-x_{j}^{1})\cdot \alpha ,x_{j}^{3}-(x_{j}^{3}-x_{j}^{2})\cdot \alpha \right]\\ +\left[q_{j}^{1}+(q_{j}^{2}-q_{j}^{1})\cdot \alpha ,q_{j}^{3}-(q_{j}^{3}-q_{j}^{2})\cdot \alpha \right] \end{array}}$$ (16)

Subjective to:

$$\begin{array}{l} \sum_{j=1}^{m}\sum_{i=1}^{n}\left[a_{ij}^{1}+(a_{ij}^{2}-a_{ij}^{1})\cdot \gamma ,a_{ij}^{3}-(a_{ij}^{3}-a_{ij}^{2})\cdot \gamma \right]\cdot \left[x_{j}^{1}+(x_{j}^{2}-x_{j}^{1})\cdot \gamma ,x_{j}^{3}-(x_{j}^{3}-x_{j}^{2})\cdot \gamma \right]\\ \leq \left[b_{j}^{1}+(b_{j}^{2}-b_{j}^{1})\cdot \gamma ,b_{j}^{3}-(b_{j}^{3}-b_{j}^{2})\cdot \gamma \right],\;i=1,2,\ldots ,m \end{array}$$ (17)

$$x_{j}^{2}-x_{j}^{1}\geq 0,x_{j}^{3}-x_{j}^{2}\geq 0,\;j=1,2,\ldots ,n$$ (18)

Based on the arithmetic operations on fuzzy numbers [38], the FFFPM can be simplified into an equivalent bi-objective programming model, which represented as following:

Bi-level fractional programming:

$$Max\tilde{Z}=\left[\begin{array}{l} \frac{\sum_{j=1}^{m}\left(c_{j}^{1}+(c_{j}^{2}-c_{j}^{1})\cdot \alpha )\cdot (x_{j}^{1}+(x_{j}^{2}-c_{j}^{1})\cdot \alpha \right)+(p_{j}^{1}+(p_{j}^{2}-p_{j}^{1})\cdot \alpha )}{\sum_{j=1}^{m}\left(d_{j}^{3}-(d_{j}^{3}-d_{j}^{2})\cdot \alpha )\cdot (x_{j}^{3}-(x_{j}^{3}-x_{j}^{2})\cdot \alpha \right)+(q_{j}^{3}-(q_{j}^{3}-q_{j}^{2})\cdot \alpha )},\\ \frac{\sum_{j=1}^{m}\left(c_{j}^{3}-(c_{j}^{3}-c_{j}^{2})\cdot \alpha )\cdot (x_{j}^{3}-(x_{j}^{3}-x_{j}^{2})\cdot \alpha \right)+(p_{j}^{3}-(p_{j}^{3}-p_{j}^{2})\cdot \alpha )}{\sum_{j=1}^{m}\left(d_{j}^{1}+(d_{j}^{2}-d_{j}^{1})\cdot \alpha )\cdot (x_{j}^{1}+(x_{j}^{2}-x_{j}^{1})\cdot \alpha \right)+(q_{j}^{1}+(q_{j}^{2}-q_{j}^{1})\cdot \alpha )} \end{array}\right]$$ (19)

Subjective to:

$$\sum_{j=1}^{n}\sum_{i=1}^{m}(a_{ij}^{1}+(a_{ij}^{2}-a_{ij}^{1})\cdot \gamma )\cdot (x_{j}^{1}+(x_{j}^{2}-x_{j}^{1})\cdot \gamma )\leq b_{j}^{1}+(b_{j}^{2}-b_{j}^{1})\cdot \gamma$$ (20)

$$\sum_{j=1}^{n}\sum_{i=1}^{m}(a_{ij}^{3}-(a_{ij}^{3}-a_{ij}^{2})\cdot \gamma )\cdot (x_{j}^{3}-(x_{j}^{3}-x_{j}^{2})\cdot \gamma )\leq b_{j}^{3}-(b_{j}^{3}-b_{j}^{2})\cdot \gamma$$ (21)

$$x_{j}^{2}-x_{j}^{1}\geq 0,x_{j}^{3}-x_{j}^{2}\geq 0,\;i=1,2,\ldots ,m,j=1,2,\ldots ,n.$$ (22)

Obtain the lower bound $Z_{\alpha }^{L}(x)$ of the objective value for different *α*-cut level, belonging to (0, 1], by formulating the following model.

Lower level fractional programming:

$$MaxZ_{ar}^{L}=\frac{\sum_{j=1}^{m}\left(c_{j}^{1}+(c_{j}^{2}-c_{j}^{1})\cdot \alpha )\cdot (x_{j}^{1}+(x_{j}^{2}-c_{j}^{1})\cdot \alpha \right)+(p_{j}^{1}+(p_{j}^{2}-p_{j}^{1})\cdot \alpha )}{\sum_{j=1}^{m}\left(d_{j}^{3}-(d_{j}^{3}-d_{j}^{2})\cdot \alpha )\cdot (x_{j}^{3}-(x_{j}^{3}-x_{j}^{2})\cdot \alpha \right)+(q_{j}^{3}-(q_{j}^{3}-q_{j}^{2})\cdot \alpha )}$$ (23)

Subjective to

$$\sum_{j=1}^{n}\sum_{i=1}^{m}(a_{ij}^{1}+(a_{ij}^{2}-a_{ij}^{1})\cdot \alpha )\cdot (x_{j}^{1}+(x_{j}^{2}-x_{j}^{1})\cdot \alpha )\leq b_{j}^{1}+(b_{j}^{2}-b_{j}^{1})\cdot \alpha$$ (24)

$$\sum_{j=1}^{n}\sum_{i=1}^{m}(a_{ij}^{3}-(a_{ij}^{3}-a_{ij}^{2})\cdot \alpha )\cdot (x_{j}^{3}-(x_{j}^{3}-x_{j}^{2})\cdot \alpha )\leq b_{j}^{3}-(b_{j}^{3}-b_{j}^{2})\cdot \alpha$$ (25)

$$x_{j}^{2}-x_{j}^{1}\geq 0,x_{j}^{3}-x_{j}^{2}\geq 0\;i=1,2,\ldots ,m,\;j=1,2,\ldots ,n$$ (26)

Then, obtain the upper bound $Z_{\alpha }^{U}(x)$ of the objective value for different *α*-cut levels by formulating the following model.

Upper level fractional programming:

$$MaxZ_{ar}^{U}=\frac{\sum_{j=1}^{m}\left(c_{j}^{3}-(c_{j}^{3}-c_{j}^{2})\cdot \alpha )\cdot (x_{j}^{3}-(x_{j}^{3}-x_{j}^{2})\cdot \alpha \right)+(p_{j}^{3}-(p_{j}^{3}-p_{j}^{2})\cdot \alpha )}{\sum_{j=1}^{m}\left(d_{j}^{1}+(d_{j}^{2}-d_{j}^{1})\cdot \alpha )\cdot (x_{j}^{1}+(x_{j}^{2}-x_{j}^{1})\cdot \alpha \right)+(q_{j}^{1}+(q_{j}^{2}-q_{j}^{1})\cdot \alpha )}$$ (27)

Subject to

$$\sum_{j=1}^{n}\sum_{i=1}^{m}(a_{ij}^{1}+(a_{ij}^{2}-a_{ij}^{1})\cdot \alpha )\cdot (x_{j}^{1}+(x_{j}^{2}-x_{j}^{1})\cdot \alpha )\leq b_{j}^{1}+(b_{j}^{2}-b_{j}^{1})\cdot \alpha$$ (28)

$$\sum_{j=1}^{n}\sum_{i=1}^{m}(a_{ij}^{3}-(a_{ij}^{3}-a_{ij}^{2})\cdot \alpha )\cdot (x_{j}^{3}-(x_{j}^{3}-x_{j}^{2})\cdot \alpha )\leq b_{j}^{3}-(b_{j}^{3}-b_{j}^{2})\cdot \alpha$$ (29)

$$x_{j}^{2}-x_{j}^{1}\geq 0,\;x_{j}^{3}-x_{j}^{2}\geq 0\;i=1,2,\ldots ,m,\;j=1,2,\ldots ,n$$ (30)

Solving the above lower level and upper level fractional programming model by the linear fractional programming method which presented in section 2.1.

And then evaluate $L\left(Z\right)={\left[Z_{ar}^{L}\right]}^{-1}$ and $R\left(Z\right)={\left[Z_{ar}^{U}\right]}^{-1}$, respectively.

Therefore, the steps of solving the FFFPM are as follows:

1. Build the original FFFPM [Eqs (10)–(12)].
2. Convert the Eqs (10)–(12) into bi-level fractional programming Eqs (19) and 20) based on the fuzzy set theory.
3. Transform the bi-level fractional programming Eqs (19) and (20) into lower level fractional programming model Eqs. (23)–(26) and upper level fractional programming model Eqs (27)–(30), respectively.
4. Solve the lower and upper level fractional programming model Eqs (23)–(26) and Eqs (27)–(30) under different *α*-cut level value by the linear fractional programming method, respectively.
5. Get the solutions under different *α*-cut level.

## Application

### Study area

**Fig 2:**
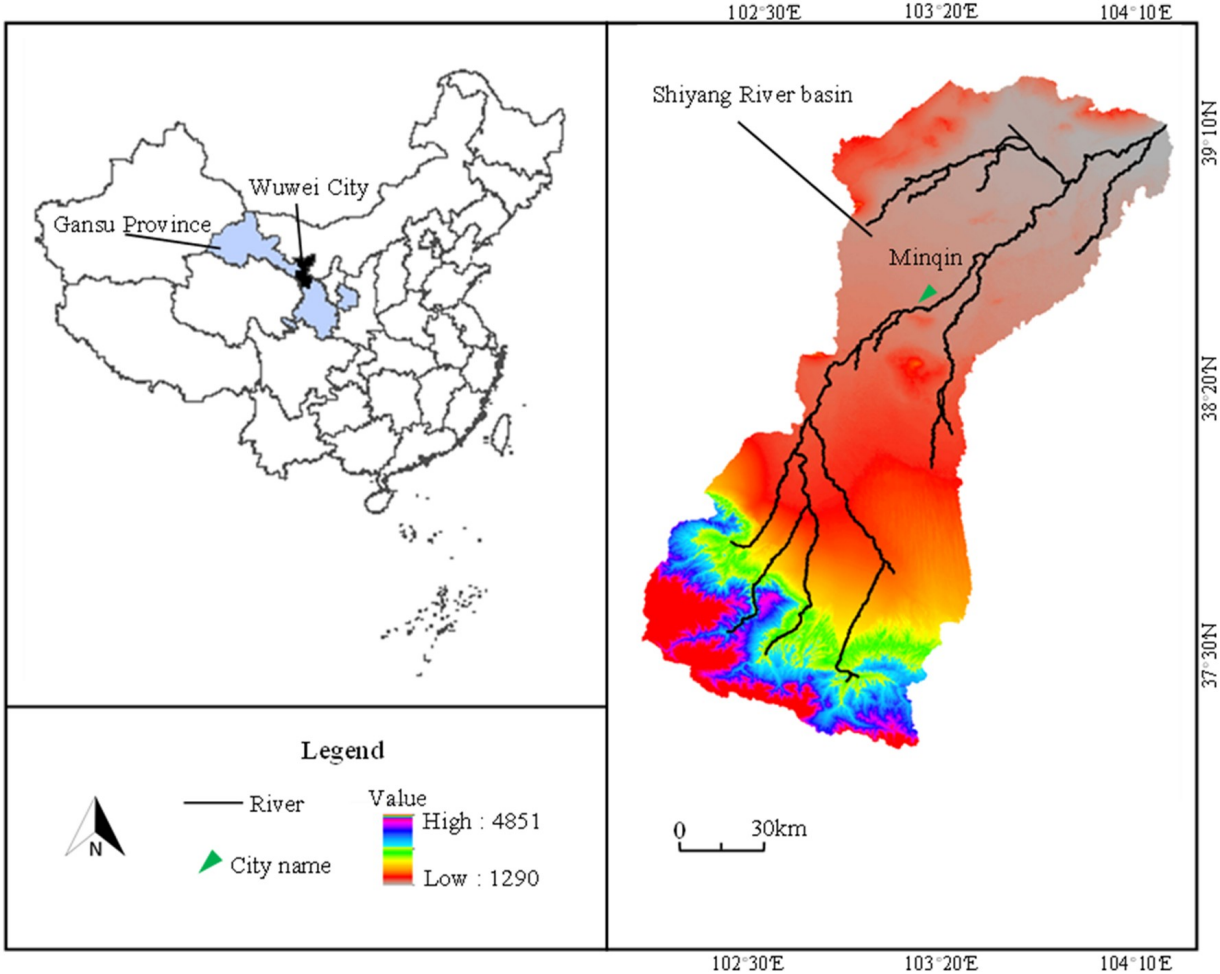
Study area.

The study area selected Minqin county (101°49′~104°12′E, 38°03′28′N), located in Gansu Province, which belongs to arid and semi-arid regions of Northwest China (Fig 2) [39]. And the study area, which is surrounded by Tengger desert and Badain Jaran desert, also located in downstream of Shiyang river basin and east of Hexi corridor [40]. Minqin is one of the most arid areas in China whose annual rainfall is about 113 mm, while the annual evapotranspiration is about 2644 mm [41].

The main water supply of Minqin are surface water and groundwater. Recently, the runoff of Shiyang river is gradually decreasing; while, the water consumption of upstream is gradually increasing [42]. Therefore, the surface water supply of Minqin has significantly decrease which aggravated the shortage of water resources. Moreover, due to the above problem, the exploitation of groundwater has increased significantly which caused serious ecological environment problems [43]. At present, in order to repair ecological environment, the exploitation of groundwater is severely restricted by the local government. However, agriculture is the biggest water consumer which belongs to heavily water consumption industry when facing more and more serious water scarcity [44]. For example, irrigation is the largest water consumption which even accounts of 88.28% of the total water consumption of Minqin [15].

Therefore, optimization allocation of irrigation water resources is badly needed for Minqin, especially aims to maximum output of unit water resources. The main crops of Minqin are wheat, corn, cotton and seed watermelon, respectively, which has been selected as study crops. Moreover, in order to obtain relatively accurate yield of study crops, linear crop production functions [45], presented in Table 1, were selected instead of yield per unit area. In addition, the surface water supply of Minqin has been controlled by Hongyashan reservoir which built in 1958. As a result, the original random characteristics of runoff has been destroyed. Based on the analysis, the surface water supply has fuzzy characteristics instead of random characteristics. Therefore, this paper established a FFFPM by taking the above problems under consideration and considering the multiple fuzzy uncertainties in the collected data. The irrigation water can be optimized based on the optimal schemes of the developed f FFFPM.

**Table 1:** The linear production function of different crops.

Crops	Water production function
Wheat	Y = -461.78+1.4518ET
Corn	Y = -405.14+1.5520ET
Cotton	Y = -112.61+0.3720ET
Seed watermelon	Y = -1308.5+0.4875ET

Notes: Y: yield of crop per unit area (kg/hm^2^); ET: water distribution per unit area (m^3^)

### Model building

Based on the above analysis, a FFFPM was established for irrigation water resources optimization allocation under multiple uncertainties. The objective of the established model was maximization economic benefit per unit of water resources based on the above analysis.

The formulation of the proposed model is presented below.

Objective function:

Maximization of economic benefit per unit of water resources

$$\text{max}=\frac{\sum_{i=1}^{4}\left(Y_{i}\cdot {\tilde{A}}_{i}\cdot {\tilde{P}}_{i}-\sum_{j=1}^{2}C_{j}\cdot {\tilde{A}}_{i}\cdot W_{ij}\right)}{\sum_{i=1}^{4}\sum_{j=1}^{2}{\tilde{A}}_{i}\cdot W_{ij}/\eta }$$ (31)

Subject to:

Surface water constraint

$$\sum_{i=1}^{4}\sum_{j=1}^{1}{\tilde{A}}_{i}\cdot W_{ij}\cdot \eta \leq \tilde{S}W$$ (32)

Groundwater constraint

$$\sum_{i=1}^{4}\sum_{j=2}^{2}{\tilde{A}}_{i}\cdot W_{ij}\cdot \eta \leq \tilde{G}W$$ (33)

Food security constraint

$$\sum_{i=1}^{2}Y_{i}\cdot {\tilde{A}}_{i}\geq FDP\cdot TPR$$ (34)

Water demand constraint

$$\sum_{j=1}^{2}W_{ij}\geq ET_{\text{min}i}$$ (35)

$$\sum_{j=1}^{2}W_{ij}\leq ET_{\text{max}i}$$ (36)

No-negative constraint

$$W_{ij}\geq 0$$ (37)

Where:

*i* is the crop index (1 = wheat, 2 = corn, 3 = cotton, 4 = seed watermelon);

*j* is the water source index (1 = surface water resources, 2 = groundwater resources);

*Y*_*i*_: Linear crop production function of crop i, which has been presented in Table 1 (t/ha);

${\tilde{A}}_{i}$: Irrigation area of crop i (10^4^ ha) (Fuzzy parameter);

${\tilde{P}}_{i}$: Price of crop i (¥/kg) (Fuzzy parameter);

*C*_*j*_: Water cost of water source j (¥/m^3^);

W_*ij*_: Water supply for crop i from water sources j (m^3^);

*η*: Irrigation water use efficiency of study area;

$\tilde{S}W$: Water supply of surface water (10^4^ m^3^) (Fuzzy parameter);

$\tilde{G}W$: Water supply of groundwater (10^4^ m^3^) (Fuzzy parameter);

*FDP*: Food demand per capita (t/p);

*TPR*: Population of the study area (10^4^ p);

*ET*_min*i*_: Minimum water demand of crop i (m^3^);

*ET*_max*i*_: Maximum water demand of crop i (m^3^);

In the established model, maximum economic benefit of per unit water resources was made as objective. Moreover, in reality, irrigation system, which involves multiple uncertainties, is a relatively complex system, such as irrigation area and price of different crops etc. In addition, the random characteristics of runoff has been destroyed since the Hongyashan reservoir was built. Thus, in this paper, maximum supply of surface water resources has fuzzy characteristics instead of random characteristics.

Tables 2 and 3 present the main parameters of the established model. The data in the table were collected through field investigations, the Water Resources Bulletin of Minqin County etc.

**Table 2:** Available water supply and water cost of surface and underground water.

Water	Available water resources (10^4^ m^3^)	Water cost (¥/m^3^)
Surface water	(10124, 10537, 16134)	0.12
Underground water	(9830, 10750, 12100)	0.20

**Table 3:** The rest parameters of the developed model.

Crops	Area (10^4^ hm^2^)	Price (¥/kg)	MaxET (m^3^)	MinET (m^3^)
Wheat	(3354.75, 3802.05, 4473.00)	(2.00, 2.40, 2.70)	5829.00	3900.00
Corn	(7235.25, 8199.95, 9647.00)	(2.20, 2.50, 3.00)	9304.50	7500.00
Cotton	(5664.75, 6420.05, 7553.00)	(15.10, 15.60, 16.40)	4303.50	3300.00
Seed watermelon	(1740.00, 1972.00, 2320.00)	(8.00, 8.50, 9.00)	2883.00	1350.00

## Result analysis and discussion

**Fig 3:**
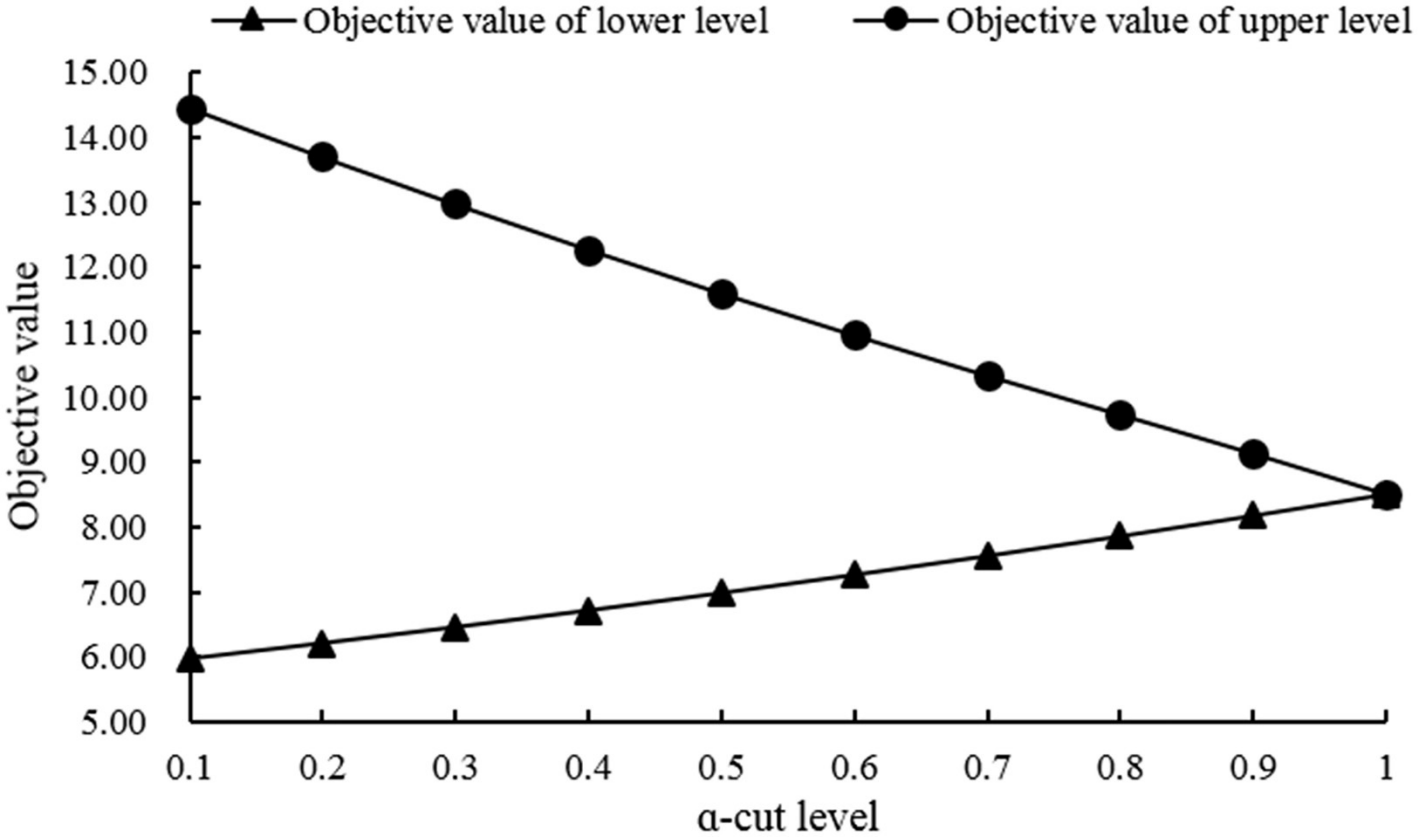
Optimized objective value under different *α*-cut levels.

In this paper, eleven *α*-cut levels were chosen by the developed model, including 0.1, 0.2, 0.3, 0.4, 0.5, 0.6, 0.7, 0.8, 0.9, 1. Fig 3 represents the optimized objective value of lower and upper level under different *α*-cut levels, respectively. From the Fig 3, the optimized objective value would vary under multiple uncertainties. As *α*-cut levels increase, the objective value of upper level would decrease; while, as *α*-cut levels increase, the objective value of lower level would increase. For example, the optimized objective value of upper level would vary from 14.95 (*α* = 0) to 8.51 (*α* = 1); while, the optimized value of lower level would vary from 5.91 (*α* = 0) to 8.51 (*α* = 1). In addition, it also represents the irrigation water use efficiency would vary as *α*-cut level changed because objective function means maximizing the economic benefit of per unit irrigation water resources.

**Fig 4:**
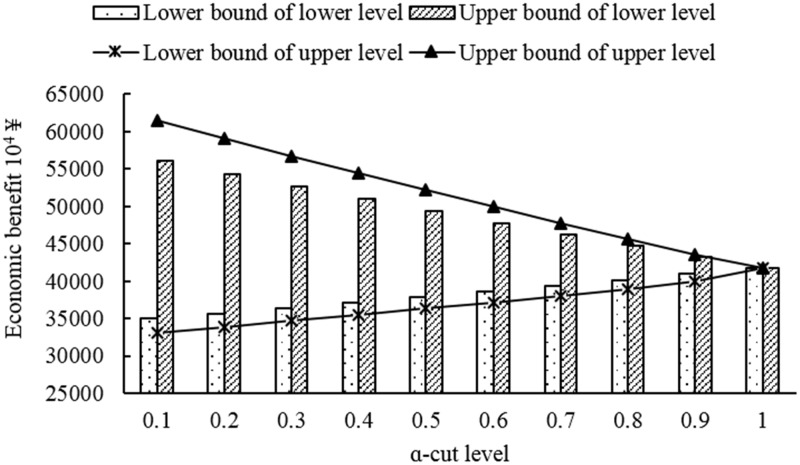
Total economic benefit under different *α*-cut levels.

Fig 4 represents the total economic benefit under different *α*-cut level. From Fig 4, when facing the upper level, the upper bound of economic benefit would decrease as *α*-cut level increased; while the lower bound of economic benefit would increase as *α*-cut level increased. When facing the lower level, they have the same trend as they in upper level. For example, upper bound of economic benefit would decrease from 61496.59×10^4^ ¥ (*α* = 0.1) to 41759.69×10^4^ ¥ (*α* = 1); while lower bound of economic benefit would increase from 33069.75 ×10^4^ ¥ (*α* = 0.1) to 41759.69 ×10^4^ ¥ (*α* = 0.1), when facing the upper level. Moreover, Fig 4 also shows that the upper bound economic benefit in upper level was higher than it in lower level under different *α*-cut levels except *α* = 1; while the upper bound economic benefit in upper level was lower than it in the lower level.

When it comes to the yield of different crops, the four crop’s yield, presented in Table 4, have roughly similar trend as total economic benefit trend in Fig 4, especially the trend of cotton yield. Compared the yield among four crops, the yield, lower bound of lower and upper level, was the same in wheat, corn and seed watermelon; while yield of cotton, lower bound of lower level, is higher than it in lower bound of upper level, which shows the same trend of total economic benefit of lower bound in lower level presented in Fig 4. According to the rule of optimizing irrigation water resources, irrigation water resources should be satisfied the minimum water demand of crops and food security constraint, firstly. And then the rest of irrigation water resources is assigned to the crops, which have relatively high yield or high economic benefit. Moreover, the developed model has the characteristic of focusing on minima and maxima. Therefore, in the lower level, the irrigation water resource was firstly satisfied the minimum water demand of wheat, corn, cotton and seed watermelon. And the rest of water was assigned to the cotton, which has characteristic of higher economic benefit than other crops. In addition, because of the characteristic of the developed model, the parameters with interval characteristic of upper level is higher than the lower level. Thus, the result of upper level pays more attention to finding the minima and maxima compared with the lower level. Therefore, the yield of lower bound in the upper level would lower than it in the lower bound. And the above analysis also explains why the total economic benefit of the lower bound in upper level is lower than it in the lower level.

**Table 4:** The yield of crops under different *α*-cut levels.

	Wheat (10^4^ kg)		Corn (10^4^ kg)		Cotton (10^4^ kg)		Seed watermelon (10^4^ kg)	
	Lower level	Upper level	Lower level	Upper level	Lower level	Upper level	Lower level	Upper level
0.1	(1766.75, 2289.80)	(1766.75,2289.80)	(8237.08,10675.70)	(8237.08,12131.97)	(771.62,1000.07)	(640.04,1079.47)	(346.76,449.57)	(346.76,449.41)
0.2	(1790, 2254.93)	(1790, 2254.93)	(8345.47,10513.12)	(8345.47,11856.22)	(771.96, 972.47)	(648.46,1035.68)	(351.32,442.57)	(351.32442.57)
0.3	(1813.24, 2220.06)	(1813.24, 2220.06)	(8453.85,10350.55)	(8453.85,11576.94)	(772.68,946.06)	(656.88,992.74)	(355.88,435.73)	(355.88,435.73)
0.4	(1836.49, 2185.19)	(1836.49, 2185.19)	(8562.23,10187.97)	(8562.2311294.13)	(773.81,920.74)	(665.30,950.65)	(360.44,428.88)	(360.44428.88)
0.5	(1859.74, 2150.32)	(1859.74, 2150.32)	(8670.62,10025.40)	(8670.62,11007.79)	(775.36,896.51)	(673.72,909.39)	(365.01,422.04)	(365.01,422.04)
0.6	(1882.98, 2115.45)	(1882.98, 2115.45)	(8779.00,9862.83)	(8779.00,10517.01)	(777.36,873.33)	(682.14,868.99)	(369.57,415.19)	(369.57,478.31)
0.7	(1906.23,2080.58)	(1906.23,2080.58)	(8887.38,9700.25)	(8887.38,10276.33)	(779.83,851.16)	(690.56,829.43)	(374.13,408.35)	(374.13,454.90)
0.8	(1929.48, 2045.71)	(1929.48, 2061.87)	(8995.76,9537.68)	(8995.76,10013.18)	(782.79,829.94)	(698.99,790.72)	(378.69,401.51)	(378.69,432.02)
0.9	(1952.72, 2010.84)	(1952.72, 2119.14)	(9104.15,9375.10)	(9104.15,9608.80)	(786.27,809.67)	(720.16,765.98)	(383.26,394.66)	(383.26,409.66)
1	(1975.97, 1975.97)	(1975.97, 1975.97)	(9212.53,9153)	(9212.53,9212.53)	(790.29,790.29)	(790.29,790.29)	(387.82,387.82)	(387.82,387.82)

**Fig 5:**
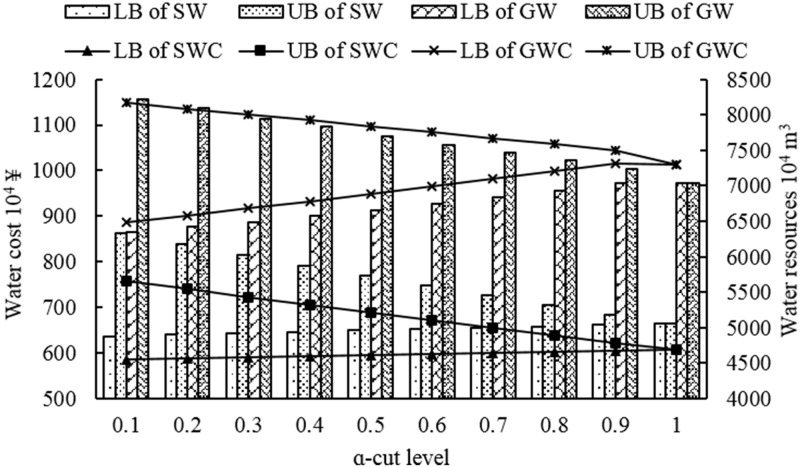
Water cost and water resources consumption under different *α*-cut levels of the lower level.

**Fig 6:**
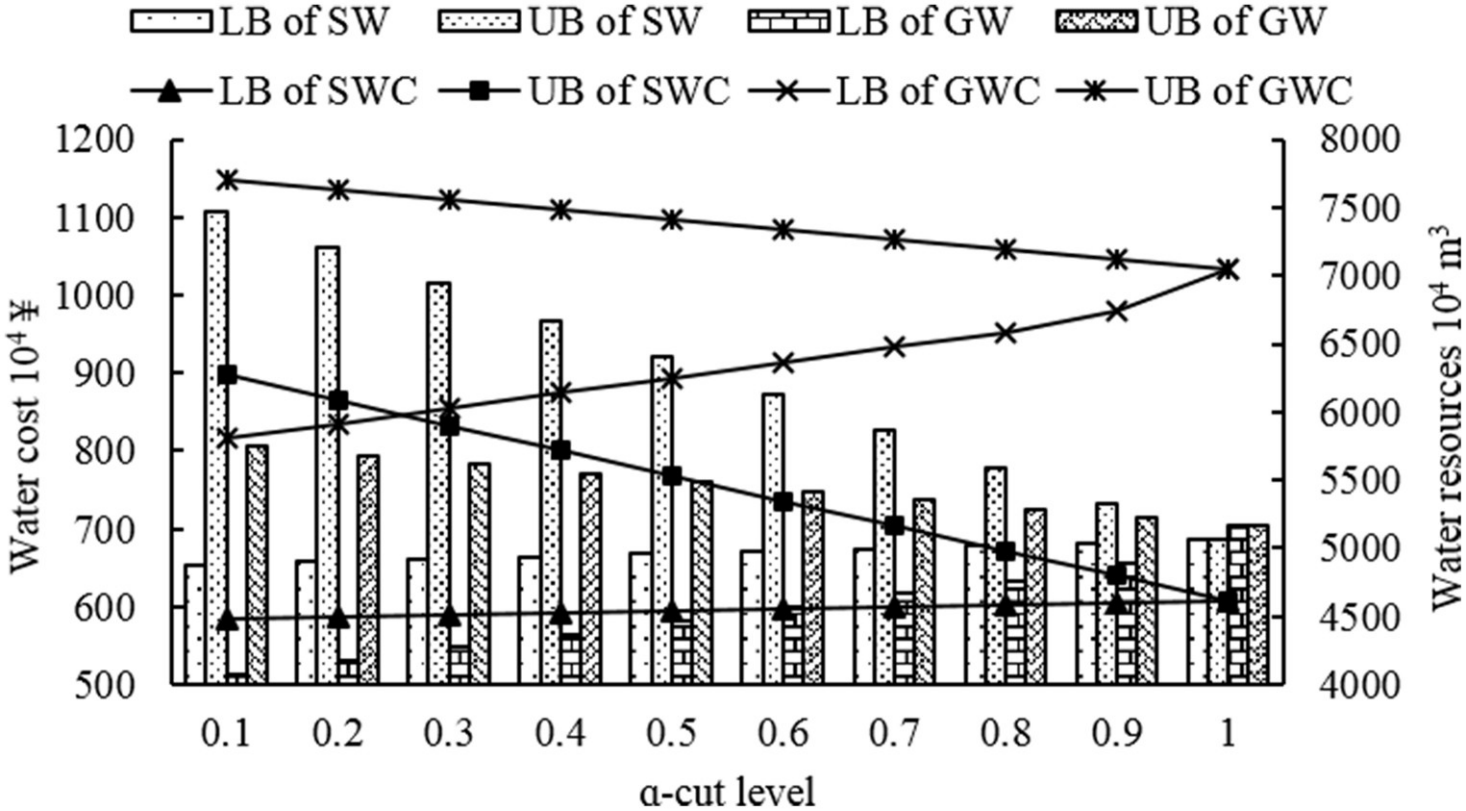
Water cost and water resources consumption under different *α*-cut levels of upper level.

Figs 5 and 6 represent the water cost and water resources consumption of surface water and underground water of lower and upper level under different *α*-cut levels, respectively. From the Figs 5 and 6, whether water cost and water resources consumption would vary as the *α*-cut levels changed. And lower bound of water cost and water resources consumption, which from both lower and upper level, would increase as *α*-cut levels increased; while, the upper bound of water cost and water resources consumption would decrease as *α*-cut levels increased. Moreover, the underground water cost was higher than surface water cost under the same bound of the same *α*-cut level whether it belongs to upper level or lower level. Moreover, although the surface water consumption was much higher than underground water consumption, the underground water cost was still much higher than surface water consumption at the same bound of the same *α*-cut level, which indicated that underground water unit price is the main reason for the above phenomenon. Compared with Figs 5 and 6, it also shows that the underground water consumption is higher than surface water consumption in the lower level; while, when it comes to the upper level, it represented just the opposite. The reason is that although the objective is maximization of economic benefit of per unit water resources, the lower level model, which was generated during the transformation of original model, focused on the lower bound of the original model actually. That is, the objective of the lower level model has been transformed into aiming at minimization lower bound value of economic benefit of per unit water resources instead of maximization of it. Therefore, it would represent the above phenomenon when they compared with each other.

**Fig 7:**
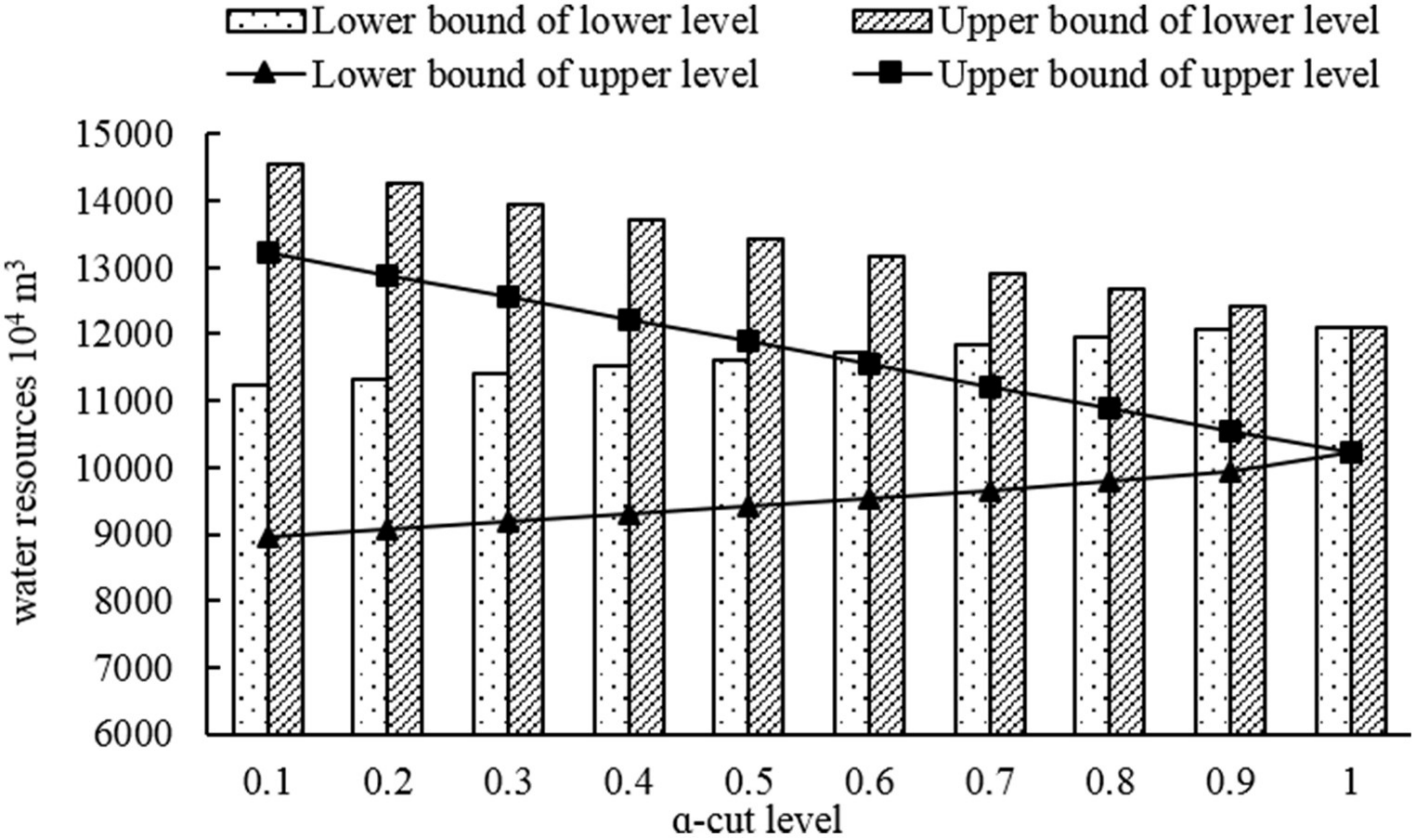
Total water resources consumption under different *α*-cut levels.

Fig 7 represents the total water resources consumption of lower and upper level under different *α*-cut levels. From the Fig 7, the total water resources consumption in lower level was higher than it in the upper level under each *α*-cut level whether the upper bound or the lower bound. When put it with the Fig 4, it could find that the total water resources consumption has great difference under each *α*-cut level whether lower bound of lower and upper level or upper bound of lower and upper level; while the total economic benefit of upper bound in upper level is higher than it of upper bound of lower level, and the difference was small when facing the lower level. And it also represented that why the economic benefit of per unit water resources in upper level is higher than that in the lower level, which has been represented in the Fig 4.

In addition, in order to deal with the multiple fuzzy uncertainties, which exists not only in objective function but also constraints, the FFFPM was established. In this paper, in order to solve developed model, the fuzzy theory was introduced in which triangular ones was selected as fuzzy membership functions. Based on the characteristics of the triangular membership function, the fuzzy possibility of the occurrence of events would increase as the *α*-cut increased; while the fuzzification would weakens as *α*-cut increased. Therefore, the gap between upper bound and lower bound, of which the optimized objective value, yield, economic and so on, was narrow when *α* = 1, and wide when *α* = 0. This is also why there are no difference between upper bound and lower bound when *α* = 1.

Based on the above analysis, the developed FFFPM has significant and positive influence on the optimization allocation of irrigation water resources. The developed model can not only optimize the irrigation water resources but also focus on water use efficiency, which has great advantage over the previous studies. And it has great positive influences to the sustainable development of Minqin county by aiming at maximizing water use efficiency, which made the maximization of economic benefit of unit water resources as objective. From the obtained results, the optimized results would vary under different *α*-cut levels. According to the results, irrigation water resources should be satisfied the minimum water demand of crops and food security constraint, firstly. And then the rest of irrigation assigned to the crops, which have relatively high yield or high economic benefit, such as cotton. For example, in the lower level, the irrigation water resources firstly satisfied the minimum water demand of wheat, corn, cotton and seed watermelon. And the rest of water was assigned to the cotton, which has characteristic of higher economic benefit than other crops. In addition, benefits per unit of irrigation water resources are also vary as different *α*-cut levels. Therefore, the decision-makers should take above aspects under consideration when a sound optimized scheme is made.

Furthermore, in real-world problem, there are multiple fuzzy uncertainties in the irrigation system, such as crop areas, crops price and so on. In addition, in reality, the random characteristic of runoff in Minqin has been destroyed, which represented as fuzzy characteristics. The developed model can deal with multiple fuzzy uncertainties. Therefore, a range of optimal schemes about optimization allocation of irrigation water resources, under different *α*-cut levels, was provided by the developed model. Based on the result, as different *α*-cut levels represent different possibility level of fuzzy sets, a sound decision schemes can be provided for the decision makers from all the optimal schemes.

## Conclusion

In this paper, a FFFPM was developed to optimization allocation of irrigation water resources under multiple fuzzy uncertainties, which existed not only in constraints but also in objective function. The proposed model has the ability to deal with uncertainties expressed as fuzzy sets, and could offer a range of optimization schemes under different *α*-cut levels.

The established model was then applied to a real-would case study in Minqin county, Gansu Province, China. In this application, maximum economic benefit of unit water resources was made as objective, which presented that it focused on maximum of water use efficiency. Compared with the previous studies, the developed made has the following advantages: (a) it can not only optimize irrigation water resources but also focus on maximize water use efficiency; (b) it has ability to deal with the multiple uncertainties which exist not only constraints but also objective function; (c) it can calculate the yield of crops more accurately by introducing the linear production function instead of yield per unit area. In addition, as different *α*-cut levels represent different levels of possibility of fuzzy sets, the developed model could provide a range of optimal schemes. A sound optimal scheme, which could meet the demand of decision makers, could be identified under multiple uncertainties.

Although the developed model provided a range of optimized solutions in this study, there is still room for research extensions. For example, irrigation system is a very complex system, which may involve multiple-objectives or objectives from different levels and so on. Moreover, although a range of parameters, characterized by fuzzy uncertainty, were considered, there are may be some factors with different kinds of uncertainty which are not take under consideration. Thus, it may be a interesting topic that how to deal with a series of uncertainties or solve the multi objective under uncertainty and so on. In addition, during the operation of other optimizing management problems, the developed model can be considered when facing multiple uncertainties such as fuzzy uncertainties.

## Supporting information

S1 Table The objective value corresponding to Fig 3. (PDF)

S2 Table The total economic benefit data corresponding to Fig 4. (PDF)

S3 Table Surface and ground water cost corresponding to Figs 5 and 6. (PDF)

S4 Table Surface and ground water consumption corresponding to Figs 5 and 6. (PDF)

S5 Table Total water resources consumption corresponding to Fig 7. (PDF)
