# Supplementary material for: An inexact fractional programming model for irrigation water resources optimal allocation under multiple uncertainties
Source: PLoS One. 2019 Jun 13;14(6):e0217783. doi: 10.1371/journal.pone.0217783 (PMC6563986; doi:10.1371/journal.pone.0217783)
Supplement: S1 Table — (PDF) [file pone.0217783.s001.pdf]

Table 1. The objective value corresponding to Fig 3

α-cut level	0.1	0.2	0.3	0.4	0.5	0.6	0.7	0.8	0.9	1
Lower level objective	5.96	6.20	6.45	6.71	6.98	7.26	7.56	7.86	8.18	8.51
Upper level objective	14.45	13.70	12.97	12.27	11.60	10.96	10.34	9.74	9.14	8.51
