# Supplementary material for: An inexact fractional programming model for irrigation water resources optimal allocation under multiple uncertainties
Source: PLoS One. 2019 Jun 13;14(6):e0217783. doi: 10.1371/journal.pone.0217783 (PMC6563986; doi:10.1371/journal.pone.0217783)
Supplement: S2 Table — (PDF) [file pone.0217783.s002.pdf]

Table 2. The total economic benefit data corresponding to fig 4

α-cut level	Lower level		Upper level	
	EL (10^4 ¥)	EU (10^4 ¥)	EL (10^4 ¥)	EU (10^4 ¥)
0.1	34982.58	56042.89	33059.7532	61496.592
0.2	35674.003	54296.561	33863.1524	59093.908
0.3	36379.969	52592.183	34676.2127	56732.193
0.4	37100.808	50928.776	35498.9341	54411.774
0.5	37836.874	49305.41	36331.3166	52132.976
0.6	38588.542	47721.2	37173.3602	49902.71
0.7	39356.214	46175.305	38025.0649	47711.491
0.8	40140.318	44666.925	38886.4307	45554.239
0.9	40941.314	43195.295	39948.8687	43480.982
1	41779.828	41779.828	41759.6906	41759.691

EL: Lower bound of economic benefit; EU: Upper bound of economic benefit.
