# Supplementary material for: An inexact fractional programming model for irrigation water resources optimal allocation under multiple uncertainties
Source: PLoS One. 2019 Jun 13;14(6):e0217783. doi: 10.1371/journal.pone.0217783 (PMC6563986; doi:10.1371/journal.pone.0217783)
Supplement: S3 Table — (PDF) [file pone.0217783.s003.pdf]

Table 3. Surface and ground water cost corresponding to figs 5 and 6

α-cut level	Lower level				Upper level			
	SWCL (10^4 ¥)	SWCU (10^4 ¥)	GWCL (10^4 ¥)	GWCU (10^4 ¥)	SWCL (10^4 ¥)	SWCU (10^4 ¥)	GWCL (10^4 ¥)	GWCU (10^4 ¥)
0.1	585.52	758.87	886.26	1148.64	585.52	897.08	815.51	1148.64
0.2	587.90	740.60	901.52	1135.68	587.90	864.84	835.12	1135.68
0.3	590.28	722.71	916.99	1122.72	590.28	832.60	854.73	1122.72
0.4	592.66	705.19	932.67	1109.76	592.66	800.36	874.33	1109.76
0.5	595.04	688.01	948.58	1096.80	595.04	768.12	893.94	1096.80
0.6	597.42	671.17	964.74	1083.84	597.42	735.89	913.54	1083.84
0.7	599.79	654.65	981.14	1070.88	599.79	703.65	933.15	1070.88
0.8	602.17	638.45	997.81	1057.92	602.17	671.41	952.76	1057.92
0.9	604.55	622.54	1014.76	1044.96	604.55	639.17	979.22	1044.96
1	606.20	606.20	1012.59	1012.59	606.93	606.93	1032.00	1032.00

SWCL: Lower bound of surface water cost; SWCU: Upper bound of surface water cost; GWCL: Lower bound of groundwater cost; GWCU: Upper bound of groundwater cost
