# Supplementary material for: An inexact fractional programming model for irrigation water resources optimal allocation under multiple uncertainties
Source: PLoS One. 2019 Jun 13;14(6):e0217783. doi: 10.1371/journal.pone.0217783 (PMC6563986; doi:10.1371/journal.pone.0217783)
Supplement: S4 Table — (PDF) [file pone.0217783.s004.pdf]

Table 4. Surface and ground water consumption corresponding to figs 5 and 6

α-cut level	Lower level				Upper level			
	SWL (10^4 m^3)	SWU (10^4 m^3)	GWL (10^4 m^3)	GWU (10^4 m^3)	SWL (10^4 m^3)	SWU (10^4 m^3)	GWL (10^4 m^3)	GWU (10^4 m^3)
0.1	4879.34	6323.89	6345.63	8224.27	4879.34	7475.66	4077.57	5743.20
0.2	4899.17	6171.68	6427.20	8096.60	4899.17	7207.01	4175.60	5678.40
0.3	4918.99	6022.61	6481.85	7936.11	4918.99	6938.35	4273.63	5613.60
0.4	4938.82	5876.57	6582.84	7832.74	4938.82	6669.70	4371.66	5548.80
0.5	4958.64	5733.43	6655.72	7695.68	4958.64	6401.04	4469.69	5484.00
0.6	4978.46	5593.09	6746.20	7579.06	4978.46	6132.38	4567.72	5419.20
0.7	4998.29	5455.45	6839.19	7464.73	4998.29	5863.73	4665.75	5354.40
0.8	5018.11	5320.41	6934.83	7352.59	5018.11	5595.07	4763.78	5289.60
0.9	5037.94	5187.87	7033.26	7242.58	5037.94	5326.42	4896.08	5224.80
1	5051.69	5051.69	7037.57	7037.57	5057.76	5057.76	5160.00	5160.00

SWL: Lower bound of surface water consumption; SWU: Upper bound of surface water consumption; GWL: Lower bound of groundwater consumption; GWU: Upper bound of groundwater consumption
