# Supplementary material for: An inexact fractional programming model for irrigation water resources optimal allocation under multiple uncertainties
Source: PLoS One. 2019 Jun 13;14(6):e0217783. doi: 10.1371/journal.pone.0217783 (PMC6563986; doi:10.1371/journal.pone.0217783)
Supplement: S5 Table — (PDF) [file pone.0217783.s005.pdf]

Table 5.Total water resources consumption corresponding to fig 7

α-cut level	Lower level		Upper level	
	TWL (10^4 m^3)	TWU (10^4 m^3)	TWL (10^4 m^3)	TWU (10^4 m^3)
0.1	11224.97	14548.16	8956.91	13218.86
0.2	11326.36	14268.28	9074.77	12885.41
0.3	11400.84	13958.72	9192.62	12551.95
0.4	11521.65	13709.31	9310.47	12218.50
0.5	11614.36	13429.11	9428.33	11885.04
0.6	11724.66	13172.15	9546.18	11551.58
0.7	11837.48	12920.17	9664.04	11218.13
0.8	11952.94	12673.00	9781.89	10884.67
0.9	12071.19	12430.45	9934.02	10551.22
1	12089.26	12089.26	10217.76	10217.76

TWL: Lower bound of total water resources consumption; TWU: Upper bound of total water resources consumption
